# Haploinsufficiency of Transferrin Receptor 1 Impairs Angiogenesis with Reduced Mitochondrial Complex I in Mice with Limb Ischemia

**DOI:** 10.1038/s41598-019-49983-4

**Published:** 2019-09-20

**Authors:** Keisuke Okuno, Yoshiro Naito, Seiki Yasumura, Hisashi Sawada, Masanori Asakura, Tohru Masuyama, Masaharu Ishihara

**Affiliations:** Department of Cardiovascular and Renal Medicine, Hyogo College of Medicine, Nishinomiya, Japan

**Keywords:** Angiogenesis, Experimental models of disease, Nutrition

## Abstract

Limb ischemia (LI) is a major consequence of peripheral artery disease (PAD) with a high mortality rate. Iron is an essential mineral to maintain physiological function of multiple organs. Intracellular iron transport is regulated by transferrin receptor 1 (TfR1). Although increase in serum ferritin levels has been reported in PAD patients, the mechanism of iron metabolism in LI is still unclear. The aim of this study is to investigate whether TfR1 deletion attenuates LI formation. To generate LI, the left femoral artery of 8–10 week-old C57BL6/J male mice was ligated. Adductor muscles were harvested at 28 days after surgery to investigate iron metabolism. The level of ferritin, intracellular iron storage protein, was higher in ischemic adductor muscles compared to non-ischemic adductor muscles. Level of intracellular iron transport protein, TfR1, was decreased in ischemic adductor muscles. LI was then generated in TfR1 heterozygous deleted mice (*TfR1*^+/−^) to examine whether TfR1 contributes to the pathophysiology of LI. Laser Doppler blood flowmetry revealed that blood flow recovery was attenuated in *TfR1*^+/−^ mice compared to wild type (WT) littermates, along with decreased expression of ferritin and CD31 in ischemic adductor muscles. Since iron is used for energy production in mitochondria, we then assessed mitochondrial complexes in the ischemic adductor muscle. Of interest, expression of mitochondrial complex I, but not complexes II, III, and V in ischemic adductor muscles was significantly reduced in *TfR1*^+/−^ mice compared to WT mice. Haploinsufficiency of TfR1 attenuated angiogenesis via reduction of mitochondrial complex I in LI in mice.

## Introduction

Limb ischemia (LI) is a leading cause of cardiovascular morbidity and mortality, and affects quality of life, physical activity, and life expectancy in patients with peripheral artery disease (PAD)^1,2^. Endovascular intervention and surgical bypass are standard definitive therapies for PAD. However, PAD patients are still at high risk of limb loss and high impairment of quality of life^3^. Since non-invasive treatment is not available for LI in PAD patients to improve their quality of life and mortality, there is an urgent need to elucidate molecular mechanisms of LI for development of therapeutic strategies to prevent the progression of PAD.

Iron is an essential trace mineral in the living body, and intracellular iron metabolism is regulated by transferrin receptor 1 (TfR1)^4^. Increase in serum ferritin levels has been reported in PAD patients^5^. In cancer cells, an association between cellular iron metabolism and angiogenesis has been reported^6^. Although angiogenesis has been reported to contribute to the pathophysiology of LI, there is no consensus regarding intracellular iron metabolism in the development of LI. Thus, in the present study, we hypothesized that intracellular iron metabolism contributes to the pathophysiology of LI. The aim of this study was to investigate whether TfR1 deletion attenuates LI formation in mice.

## Results

### Increased ferritin expression in the ischemic adductor muscle of LI-induced mice

**Figure 1:**
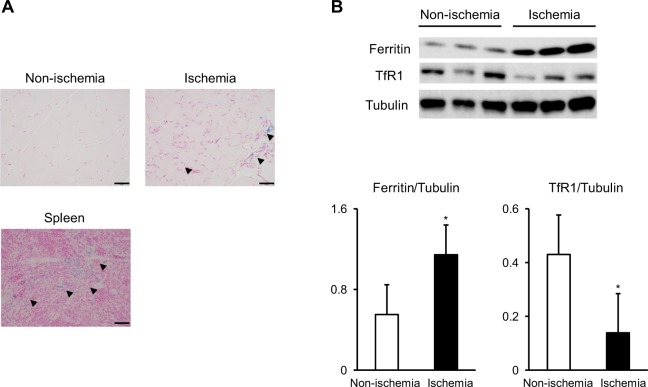
Intracellular iron metabolism in ischemic adductor muscle tissues of LI mice: (**A**) Representative images of berlin blue staining of non-ischemic and ischemic adductor muscles, and spleen sections at 28 days after the surgery. Arrow heads indicate berlin blue positive areas. (**B**) Representative western blotting image and summary densitometry graph illustrating expression of ferritin and TfR1 in non-ischemic and ischemic adductor muscles at 28 days after the surgery (n = 6 in each group). *p < 0.05 versus non-ischemic adductor muscles.

We first assessed iron content in the adductor muscle of LI-induced mice at 28 days after the surgery. In berlin blue staining, the non-ischemic adductor muscle did not show positive staining area, while partial iron deposition was observed in the ischemic adductor muscle (Fig. 1A). We next assessed the expression of cellular iron regulatory proteins in the adductor muscle of LI-induced mice at 28 days after the surgery. The level of ferritin, intracellular iron storage protein, was higher in the ischemic muscle compared to the non-ischemic muscle (Fig. 1B). Contrary to ferritin abundance, the expression of TfR1, intracellular iron import protein, was decreased in the ischemic muscle (Fig. 1B). The expression of ferroportin, intracellular iron export protein, was not significantly different between the ischemic and non-ischemic muscles (Supplementary Fig. 1A). Regarding tissue iron content, there was a trend toward an increase in the ischemic muscle compared to the non-ischemic muscle; however, it was not statistically significant between the ischemic and non-ischemic muscles (Supplementary Fig. 1B).

### Low iron condition due to iron restriction did not affect angiogenesis in the adductor muscle of LI-induced mice

**Figure 2:**
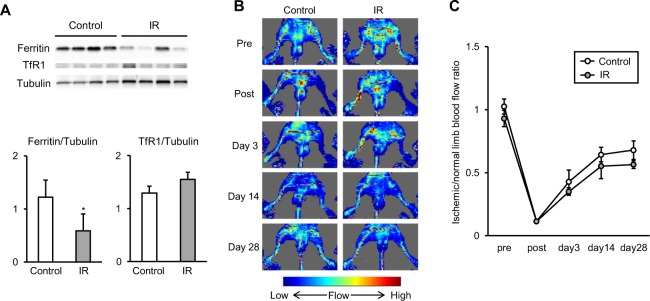
Impacts of dietary iron restriction on the development of angiogenesis in LI mice: (**A**) Representative western blotting image and summary densitometry graph illustrating expression of ferritin and TfR1 in the ischemic adductor muscle of the control and IR groups at 28 days after the surgery (n = 6 in each group). (**B**) Representative images of LDBF of mice subjected to limb ischemia in the control and IR groups. (**C**) Quantitative analysis of the ischemic/normal LDBF ratio in the control and IR groups on pre, postoperative days 0, 3, 14, and 28 (n = 11 in the control group, n = 8 in the IR group). Control, LI-induced mice fed a normal diet; IR, LI-induced mice fed an iron-restricted diet. *p < 0.05 versus the control groups.

To investigate the role of increased ferritin expression in the ischemic adductor muscle, we next performed dietary iron restriction in LI-induced mice to reduce muscular iron content. During the experiment, the feeding behavior and activity did not differ between the groups, and no mice died after the surgery. As shown in Table 1, dietary iron restriction did not affect body weight, blood hemoglobin content, and hematocrit value at 28 days after the surgery. However, mean corpuscular volume was less in the IR group compared to the control group. Ferritin expression in the ischemic adductor muscle was also decreased in the IR group compared to the control group (Fig. 2A). Meanwhile, TfR1 expression was comparable in the ischemic adductor muscle between the control and IR groups (Fig. 2A). Ferroportin expression was also not significantly different between the control and IR groups (Supplementary Fig. 2A). Regarding tissue iron content, there were no statistically significant differences in the ischemic adductor muscle between the control and IR groups (Supplementary Fig. 2B). These results indicated that dietary iron restriction induced mild low iron condition without anemia. In this condition, limb blood flow was measured sequentially, and there was no difference on limb blood flow between the control and IR groups (Fig. 2B,C). Taken together, these results suggested that low iron condition by dietary iron restriction did not affect angiogenesis in the adductor muscle of LI-induced mice.

**Table 1:** Physiological parameters in the Control and IR groups at 28 days after the surgery.

Parameter	Control	IR
n	11	8
Body weight (g)	23.9 ± 0.6	23.7 ± 0.5
Hemoglobin (g/dL)	14.6 ± 0.1	14.9 ± 0.1
Hematocrit (%)	51.2 ± 0.5	52.1 ± 0.5
MCV (µm^3^)	52.2 ± 0.8	48.6 ± 0.2*

*p < 0.05 versus the Control group. Control = LI-induced mice fed a normal diet; IR = LI-induced mice fed an iron-restricted diet; MCV = mean corpuscular volume.

### Haploinsufficiency of TfR1 inhibited revascularization in the adductor muscle of LI-induced mice

**Figure 3:**
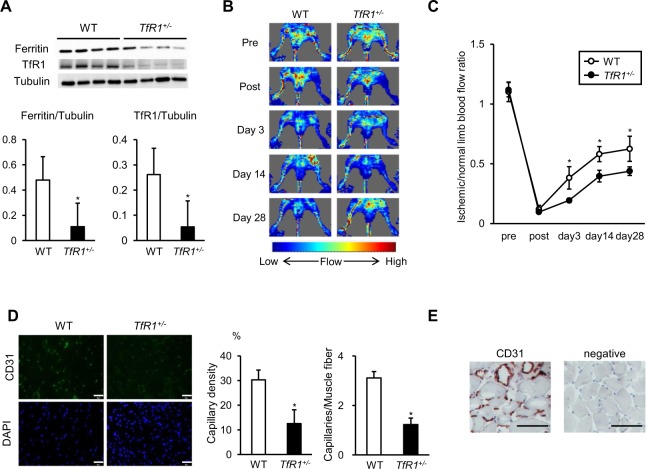
Haploinsufficiency of TfR1 on the development of angiogenesis in LI mice. (**A**) Representative western blotting image and summary densitometry graph illustrating expression of ferritin and TfR1 in the ischemic adductor muscle of WT and *TfR1*^+/−^ mice at 28 days after the surgery (n = 6 in each group). (**B**) Representative images of LDBF of WT and *TfR1*^+/−^ mice subjected to limb ischemia. (**C**) Quantitative analysis of the ischemic/normal LDBF ratio in WT and *TfR1*^+/−^ mice on pre, postoperative days 0, 3, 14, and 28 (n = 6 in WT, n = 12 in *TfR1*^+/−^). (**D**) Representative images of CD31 and DAPI staining of ischemic adductor muscle sections and quantitative analysis of the capillary density and the number of CD31-positive cells per muscle fiber of the ischemic adductor muscle in WT and *TfR1*^+/−^ mice at 28 days after the surgery (n = 6 in each group). Scale bars: 50 µm. (**E**) Representative images of anti-CD31 immunohistochemical staining and non-immune IgG (negative) staining of ischemic adductor muscle sections in WT mice at 28 days after the surgery. Scale bars: 100 µm. WT, wild-type mice; *TfR1*^+/−^, TfR1 heterozygous deleted mice. *p < 0.05 versus WT mice.

Since intracellular iron transport is regulated by TfR1^4^, LI surgery was performed in WT and *TfR1*^+/−^ mice to investigate whether TfR1 plays a role in the pathophysiology of LI. Table 2 shows physiological parameters at 28 days after LI surgery. Body weight, blood hemoglobin content, and hematocrit value were not significantly different between WT and *TfR1*^+/−^ mice, while mean corpuscular volume was less in *TfR1*^+/−^ mice than in WT mice, which was consistent with the previous reports^7,8^. The expression levels of ferritin and TfR1 in the ischemic adductor muscle were lower in *TfR1*^+/−^ mice compared to WT mice (Fig. 3A). Ferroportin expression in the ischemic adductor muscle was not significantly different between WT and *TfR1*^+/−^ mice (Supplementary Fig. 3A). Regarding tissue iron content, there were no significant differences in the ischemic adductor muscle between WT and *TfR1*^+/−^ mice (Supplementary Fig. 3B). LDBF showed that blood flow recovery in the ischemic limb was attenuated by TfR1 heterozygous deletion (Fig. 3B,C). Then, to further investigate angiogenesis in the ischemic adductor muscle, CD31 positive cells were evaluated in different groups. The abundance of CD31 positive cells in the ischemic adductor muscle was lower in *TfR1*^+/−^ mice than WT mice at 28 days after the surgery (Fig. 3D). Specificity of CD31 staining was confirmed by immunohistochemical staining (Fig. 3E). Taken together, these results indicated that heterozygous TfR1 deletion impaired angiogenesis in the ischemic adductor muscle.

**Table 2:** Physiological parameters in WT and *TfR1*^+/−^ mice at 28 days after the surgery.

Parameter	WT	*TfR1* ^+/−^
n	6	12
Body weight (g)	24.7 ± 1.0	24.9 ± 0.7
Hemoglobin (g/dL)	14.1 ± 0.4	13.3 ± 0.3
Hematocrit (%)	49.2 ± 1.6	47.2 ± 0.9
MCV (µm^3^)	51.3 ± 0.3	45.3 ± 1.1*

*p < 0.05 versus WT mice. MCV = mean corpuscular volume.

### Haploinsufficiency of TfR1 inhibited mitochondrial complex I expression in the ischemic adductor muscle of LI-induced mice

**Figure 4:**
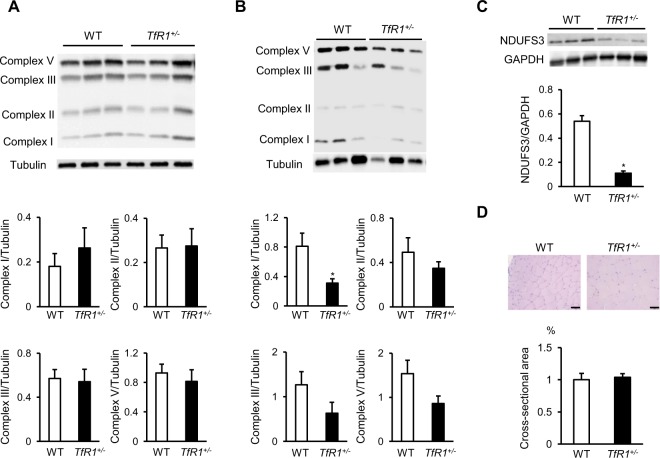
Impacts of haploinsufficiency of TfR1 in OXPHOS members expression in LI mice: Representative western blotting image and summary densitometry graph illustrating expression of OXPHOS members in the adductor muscle of WT and *TfR1*^+/−^ mice (**A**) before (n = 6 in each group) and (**B**) at 28 days after the surgery (n = 6 in each group). (**C**) Representative western blotting image and summary densitometry graph illustrating expression of NDUFS3 in the ischemic adductor muscle of WT and *TfR1*^+/−^ mice at 28 days after the surgery (n = 6 in each group). (**D**) Representative images of hematoxylin and eosin staining of ischemic adductor muscle sections and quantitative analysis of relative cross-sectional area of the ischemic adductor muscle in WT and *TfR1*^+/−^ mice at 28 days after the surgery (n = 6 in each group). WT, wild-type mice; *TfR1*^+/−^, TfR1 heterozygous deleted mice. *p < 0.05 versus WT mice.

Mitochondria are abundant in skeletal muscles and mitochondria in limb skeletal muscles are associated with the pathophysiology of LI^9^. Iron is used for energy production in mitochondria. Thus, the integrity of electron transfer chain complexes I-V in the ischemic adductor muscle was evaluated before and at 28 days after the surgery to assess the mechanism by which heterozygous deletion of TfR1 affects angiogenesis during LI. At the baseline, the expressions of all mitochondrial complexes in the adductor muscle were comparable between WT and *TfR1*^+/−^ mice (Fig. 4A). At 28 days after the surgery, expressions of mitochondrial complexes II, III, and V were not significantly different between the two groups. However, interestingly, mitochondrial complex I expression in the ischemic adductor muscle was significantly decreased in *TfR1*^+/−^ mice compared to WT mice at 28 days after the surgery (Fig. 4B). In addition, the expression of NDUFS3, a key iron-sulfur containing protein of complex I, was decreased in the ischemic adductor muscle of *TfR1*^+/−^ mice compared to WT mice (Fig. 4C). We finally evaluated the impact of TfR1 heterozygous deletion on muscle morphology. There was no significant difference in the cross-sectional area of the ischemic adductor muscle between WT and *TfR1*^+/−^ mice at 28 days after the surgery (Fig. 4D). These results indicated that haploinsufficiency of TfR1 decreased mitochondrial complex I expression in the ischemic adductor muscle in LI-induced mice.

## Discussion

In the present study, we demonstrated increased ferritin and decreased TfR1 expression in the ischemic adductor muscle of LI-induced mice. In addition, TfR1 heterozygous deletion attenuates angiogenesis with reduced expression of ferritin and mitochondrial complex I. This is the first time, to the best of our knowledge, that an association between cellular iron metabolism and angiogenesis in LI has been reported.

Several clinical studies have suggested an association between iron and PAD^10^. Serum ferritin levels in PAD patient are higher than normal subjects^5^. These observations suggested a link between iron and the pathophysiology of PAD. LI is a major consequence of PAD. Thus, in the present study, we initially hypothesized that local intracellular iron metabolism in ischemic adductor muscle tissues contributes to the pathophysiology of LI. We found that the expression of ferritin, an intracellular iron storage protein, was increased and the expression of TfR1, an intracellular iron import protein, was decreased in the ischemic adductor muscle compared to the non-ischemic adductor muscle. In addition, the expression of ferroportin, an intracellular iron export protein, was not significantly different between the ischemic and non-ischemic adductor muscles. These results suggested that local intracellular iron metabolism in ischemic adductor muscle tissues might be associated with the pathophysiology of LI.

Then, to investigate the role of increased ferritin expression in the ischemic adductor muscle, we next assessed the impact of dietary iron restriction on the development of LI in mice. In the present study, dietary iron restriction induced a reduction in ferritin expression, without significant changes of TfR1 and ferroportin expression in the ischemic adductor muscle. In this condition, quantitative analysis of limb perfusion was not statistically significant between the control and IR groups. These results suggested that reduced ferritin expression in the ischemic adductor muscle might not affect angiogenesis in LI-induced mice. A previous randomized trial has shown that reduction in body iron stores by phlebotomy did not significantly affect clinical outcomes in PAD patients^11^. Our data might be consistent with this clinical result.

TfR1 plays a key role in cellular iron transport^4^. To investigate whether TfR1 plays a role in the pathophysiology of LI, we further subjected *TfR1*^+/−^ mice to LI. Of interest, LDBF showed that blood flow recovery in the ischemic limb was attenuated in *TfR1*^+/−^ mice compared to WT mice, along with decreased expression of ferritin and TfR1 in the ischemic adductor muscle. On the other hand, ferroportin expression was not significantly different in the ischemic adductor muscle between WT and *TfR1*^+/−^ mice. Considering with the results of dietary iron restriction on LI mice, these results suggested that TfR1 in the ischemic adductor muscle contributes to the development of LI.

We have previously showed adverse roles of iron in cardiovascular diseases, including hypertension, pulmonary hypertension, chronic kidney disease, and aortic aneurysm^8,12–15^. Therefore, in the present study, we initially expected that iron exerts an adverse role in the pathophysiology of LI. However, dietary iron restriction did not affect angiogenesis in LI mice with reduced ferritin expression in the ischemic adductor muscle. In addition, we found that TfR1 heterozygous deletion attenuated angiogenesis with reduced expression of ferritin and TfR1 in the ischemic adductor muscle. Since transient withdrawal of dietary iron could be overcome from storage iron of the liver, the effect of dietary iron restriction on LI mice might be different from the results in *TfR1*^+/−^ mice. There are abundant mitochondria in skeletal muscles, and iron is used for energy production in mitochondria. A previous study has reported that mice lacking TfR1 in skeletal muscles showed incapacitated energy production in muscles^16^. Taken together, iron seems to be necessary for the ischemic adductor muscle. In the ischemic adductor muscle of LI-induced mice, ferritin expression was increased and TfR1 expression was decreased compared to the non-ischemic adductor muscle, which might show the necessity of iron for the ischemic adductor muscle.

Mitochondria are abundant in skeletal muscles, and mitochondriopathy in limb skeletal muscles is related to the pathophysiology of LI^9^. A previous study has shown that mitochondria-targeting peptide prevents limb perfusion after LI^17^. Iron is used for energy production via mitochondria. Thus, to investigate the mechanism by which heterozygous deletion of TfR1 decreases angiogenesis in LI, we finally assessed mitochondrial complex in the ischemic adductor muscle. Of note, mitochondrial complex I expression in the ischemic adductor muscle was decreased in *TfR1*^+/−^ mice compared to WT mice, but not complexes II, III, and V. These results are consistent with a previous report in which iron deficiency without anemia impaired the expression cardiac mitochondrial complex I, but not the other complexes in mice^18^. Since the expression of all mitochondrial complexes in the adductor muscle did not differ significantly between WT and *TfR1*^+/−^ mice before the surgery, these results indicated that impaired mitochondrial complex I expression after the surgery depends on heterozygous TfR1 deletion in the ischemic adductor muscle. Among the respiratory chain complexes, complex I contains the most iron-sulfur centers^19^. In the present study, we confirmed that the expression of NDUFS3, a key iron-sulfur containing protein of complex I, was also decreased in the ischemic adductor muscle of *TfR1*^+/−^ mice compared to WT mice. Mitochondrial complex I is the first electron acceptor, which is responsible for electron transport chain complexes and ATP production, affecting mitochondrial function directly^20^. Collectively, these results suggested that impaired mitochondrial complex I expression in the ischemic adductor muscle might induce impaired skeletal muscle mitochondrial respiratory capacity and attenuate angiogenesis in *TfR1*^+/−^ mice after the surgery.

The association between iron and skeletal muscle atrophy has been reported^21^, we thus evaluated the impact of TfR1 heterozygous deletion on muscle morphology. There was no significant difference in the cross-sectional area of the ischemic adductor muscle between WT and *TfR1*^+/−^ mice at 28 days after the surgery. A clinical study has shown that the cross-sectional area of the ischemic muscle was not different between healthy subjects and patients with intermittent claudication^9^. Our result might be consistent with this data. Although angiogenesis was attenuated in *TfR1*^+/−^ mice after the surgery, it is unknown whether this result is mediated by direct effects on vascular cells or indirect effects, i.e. damage in skeletal muscles. Future experimental work will define what kind of cells contributes to angiogenesis in *TfR1*^+/−^ mice after the surgery.

In conclusion, haploinsufficiency of TfR1 attenuated angiogenesis with reduced expression of ferritin and mitochondrial complex I in a mouse model of LI.

## Methods

### Animals

All of our experimental procedures were approved by the Animal Research Committee of Hyogo College of Medicine (protocol #17-006), and were performed in accordance with National Institutes of Health guidelines. Mice were housed in a temperature-controlled facility with a 12 h light/dark cycle and were provided food and water *ad libitum*.

### Mouse model of LI

To induce LI, eight to ten week old male mice were anesthetized using isoflurane, and the proximal portion of the femoral artery and the distal portion of the saphenous artery were ligated and excised. At five time points (10 min before and after the surgery as well as after 3, 14, and 28 days), Laser Doppler blood flowmetry (LDBF, Moor LDI2-IR, Moor Instruments, Inc. DE, USA) was performed to examine blood flow of left (ischemic) and right (non-ischemic) hind limbs. Blood flow was shown as the ratio of ischemic/non-ischemic hind limb blood flow to minimize artifact due to ambient light. At 28 days after the surgery, the adductor muscle and blood were collected.

#### Protocol 1

C57BL/6 J mice were purchased from CLEA Japan, Inc. (Tokyo, Japan). Mice were fed on a normal diet for 1 week and subjected to LI. To manipulate muscular iron content, LI-induced mice were provided with an iron-restricted diet (IR, n = 8) for 28 days. *Ad libitum* normal diet was used as control (Control, n = 11). The components of a normal diet consisted of 33% corn starch, 22% casein, 5% cellulose, 30% sucrose, 5% corn oil, 4% mineral mixture, and 1% vitamin mix. Mineral mixture contained 0.43% CaHPO_4_∙2H_2_O, 34.31% KH_2_PO_4_, 25.06% NaCl, 0.623% FeC_6_H_5_O_7_∙5H_2_O, 4.8764% MgSO_4_, 0.02% ZnCl_2_, 0.121% MnSO_4_∙5H_2_O, 0.156% CuSO_4_∙5H_2_O, 0.0005% KI, 29.29% CaCO_3_, 0.0025% (NH_4_)_6_M_O7_O_24_∙4H_2_O, and 5.11% microcrystalline cellulose. An iron-restricted diet was similar to a normal diet, but contained a mineral mixture free of FeC_6_H_5_O_7_∙5H_2_O, as previously described^12^.

#### Protocol 2

TfR1 heterozygous deleted (*TfR1*^+/−^) mice (C57BL/6J background) were obtained from the Jackson laboratory (#018387). Since homozygous deletion of TfR1 causes embryonic lethality due to hematopoietic defect^7^, *TfR1*^+/−^ mice were used in this study (n = 12). Non transgenic littermates were used as control (WT). LI was established in *TfR1*^+/−^ and WT mice (n = 12 and 6, respectively). Blood cell count was measured by an automatic cell count analyzer (Pentra 60 LC-5000, Horiba, Kyoto, Japan), as previously described^13^.

### Western blot analysis

The adductor muscles were homogenized using freeze crusher (SK-100, Tokken Inc., Chiba, Japan) and cell lysis buffer (#9803, Cell Signaling Technology, MA, USA). The protein concentration was determined by DC assay kit (#5000112, Bio-Rad, CA, USA). Thirty micrograms of protein homogenate was separated by SDS-PAGE and transferred onto polyvinylidene fluoride membranes. The expression levels of proteins were detected by an enhanced chemiluminescence kit (#32106, Thermo Scientific, IL, USA). Anti-ferritin (#ab75973, Abcam, UK; dilution 1:1000), anti-TfR1 (#13-6800, Invitrogen, CA, USA; dilution 1:1000), anti-ferroportin (NBP1-21502, Novus Biologicals, CO, USA; dilution 1:1000), anti-tubulin (#PM054-7, MBL, Aichi, Japan; dilution 1:1000), anti-mitochondrial total oxidative phosphorylation (OXPHOS) antibody cocktail including antibodies against C I, C II, C III, and C V (#ab110413, Abcam; dilution 1:1000), anti-NADH dehydrogenase iron-sulfur protein 3 (NDUFS3, #ab110246, Abcam; dilution 1:1000), and anti-GAPDH (#2118, Cell Signaling Technology; dilution 1:1000) antibodies were used.

### Histological analyses

Adductor muscles were quickly embedded in Tissue-Tek OCT compound (Sakura Finetechnical Co., Tokyo, Japan) and snap-frozen in liquid nitrogen. The tissues were cut into 5-µm-thick sections. Hematoxylin-eosin was performed by a standard protocol as previously described. Immunohistochemistry for CD31 were performed using a rat anti-CD31 antibody (#553370, BD Pharmingen, NJ, USA; dilution 1:1000) as a primary antibody. Primary antibody was sequentially coupled with biotin-conjugated goat anti-rat antibody (#BA-4000, Vector Laboratories, CA, USA) and visualized by VECTASTAIN Elite ABC HRP kit (#PK6100, Vector Laboratories). The staining was then revealed using a 3-amino-9-ethylcarbazole (AEC) peroxidase substrate kit (#SK4205, Vector Laboratories). Tissue sections were counterstained with hematoxylin. For immunofluorescence analysis, a rat anti-CD31 primary antibody was coupled with Alexa Fluor 488 secondary antibody (#A11006, Thermo Scientific) and nuclei were counterstained with DAPI (#H-1200, Vector Laboratories). Isotype-match control rat IgG antibody (#ab18541, Abcam) was used to examine antibody specificity. Capillary density and cross-sectional area of muscles were quantified by NIH ImageJ software. Sixteen random microscopic fields from 4 different sections in each mouse were counted (magnification, 400x) for the capillary density analysis and cross-sectional area of muscles in a blinded manner. Capillary density was expressed as CD31-positive cells per total number of DAPI-positive cells and the number of CD31-positive cells per muscle fiber. In addition, adductor muscular tissues were fixed with 4% paraformaldehyde, and embedded in paraffin. The tissues were cut into 4-µm-thick sections that were used for berlin blue staining. Berlin blue staining was performed using freshly prepared 2% potassium ferrocyanide and 2% hydrochloric acid. Spleen was used as a berlin blue positive control.

### Assessments of tissue iron content

Tissue iron content of the adductor muscle was measured by Metallo assay kit according to the manufacturer’s instructions (FE02ME, Metallogenics Co, Chiba, Japan). Iron content was then corrected to adductor muscle weight for each sample.

### Statistical analysis

Values are reported as the mean ± SEM. Normality and equal variance were assessed prior to comparison by Shapiro-Wilk and Brown-Forsythe tests, respectively, and all data passed these tests. Student’s *t-*test was performed for comparison between two groups. Repeated measures ANOVA was used for analysis for laser doppler imaging data. P value < 0.05 was considered statistically significant.

## Supplementary information

Supplementary Information
