# Supplementary material for: Haploinsufficiency of Transferrin Receptor 1 Impairs Angiogenesis with Reduced Mitochondrial Complex I in Mice with Limb Ischemia
Source: Sci Rep. 2019 Sep 20;9:13658. doi: 10.1038/s41598-019-49983-4 (PMC6754437; doi:10.1038/s41598-019-49983-4)
Supplement: Supplementary file 1 — Supplementary Information [file 41598_2019_49983_MOESM1_ESM.pdf]

Supplementary Information

Haploinsufficiency of Transferrin Receptor 1 Impairs Angiogenesis with Reduced Mitochondrial Complex I in Mice with Limb Ischemia

Keisuke Okuno, Yoshiro Naito, Seiki Yasumura, Hisashi Sawada, Masanori Asakura, Tohru Masuyama, Masaharu Ishihara

Division of Cardiovascular Medicine and Coronary Heart Disease, Department of Internal Medicine, Hyogo College of Medicine, Nishinomiya, Japan

Supplementary Figure 1

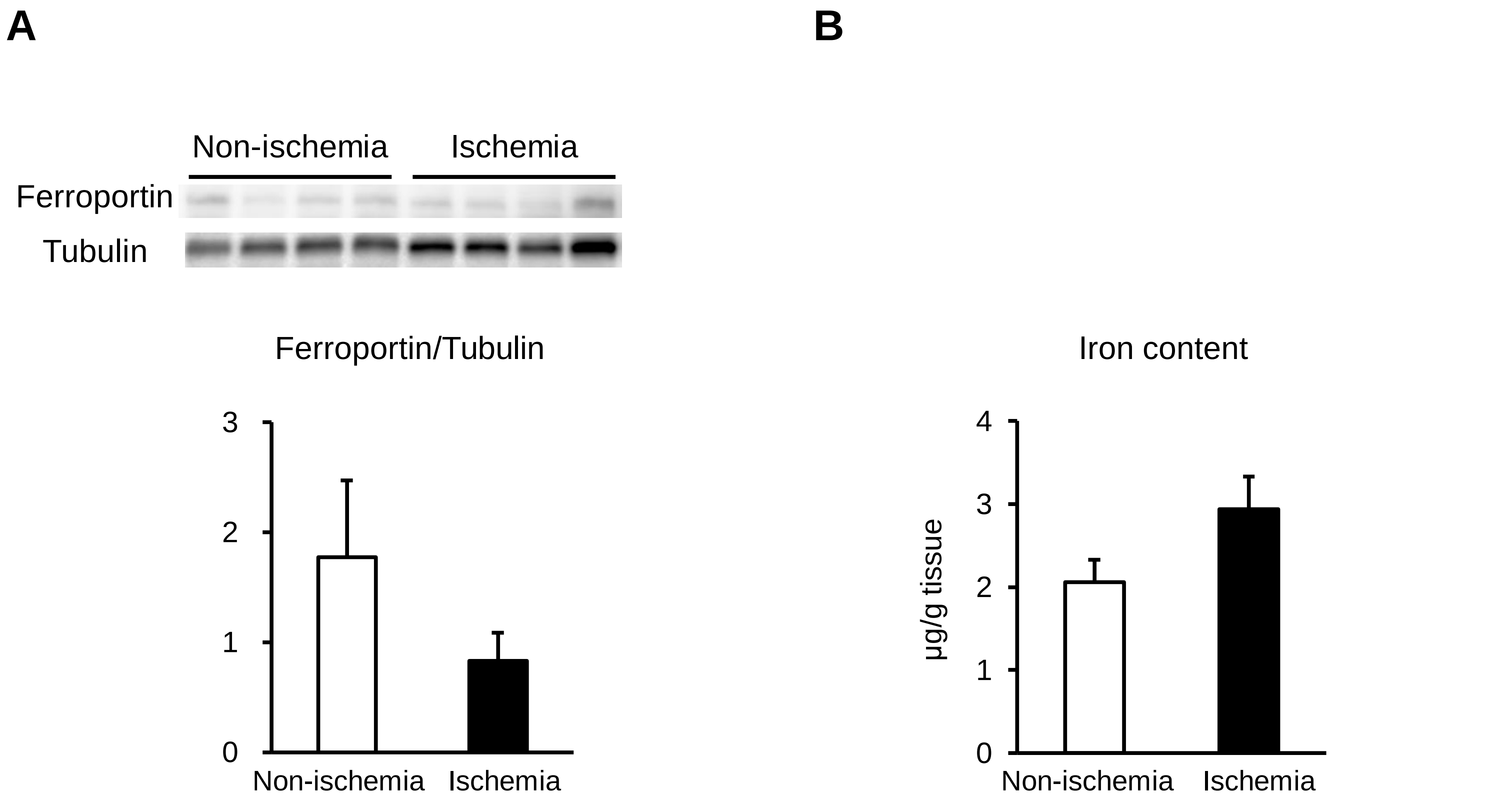

Intracellular iron metabolism in ischemic adductor muscle tissues of LI mice

(A) Representative western blotting image and summary densitometry graph illustrating expression of ferroportin and (B) iron contents in non-ischemic and ischemic adductor muscles at 28 days after the surgery.

Supplementary Figure 2

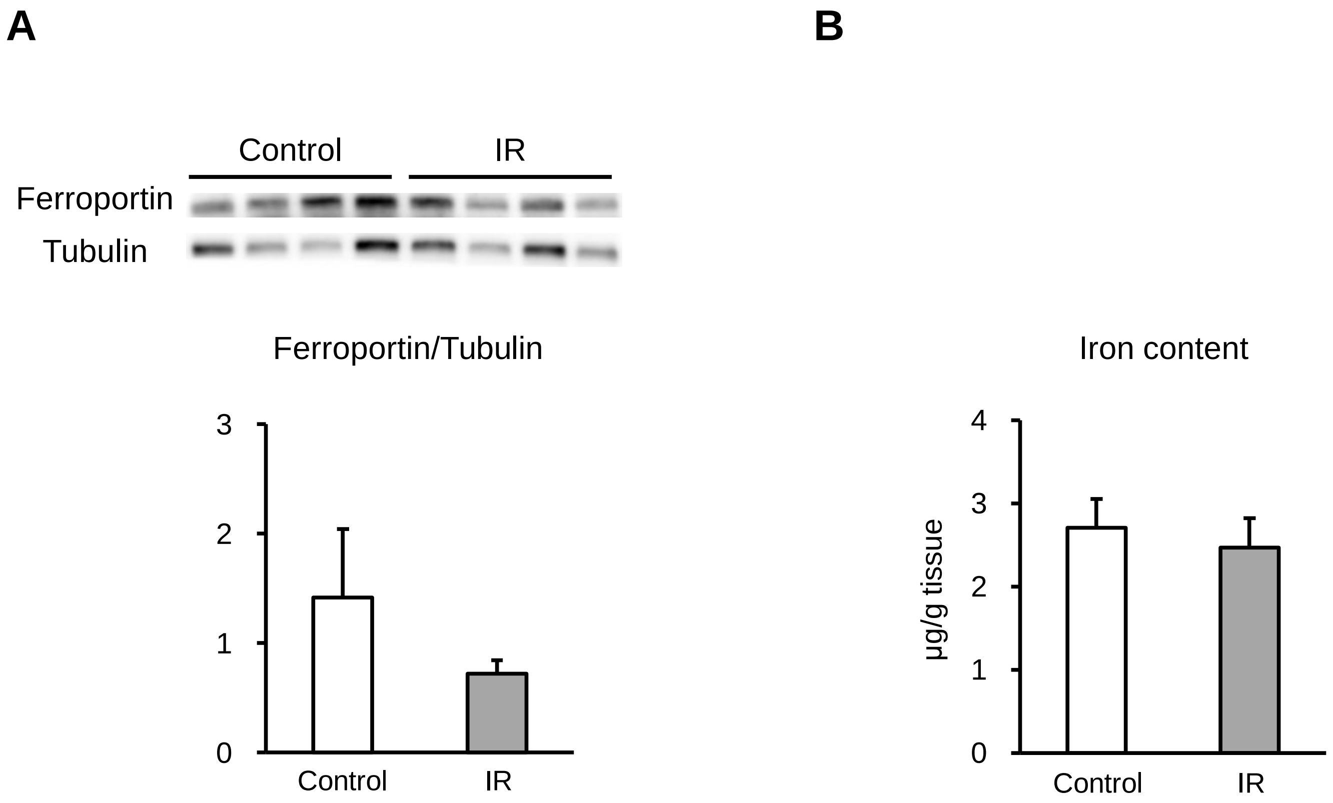

Impacts of dietary iron restriction on the development of angiogenesis in LI mice

(A) Representative western blotting image and summary densitometry graph illustrating expression of ferroportin and (B) iron contents in the ischemic adductor muscle of the control and IR groups at 28 days after the surgery.

Supplementary Figure 3

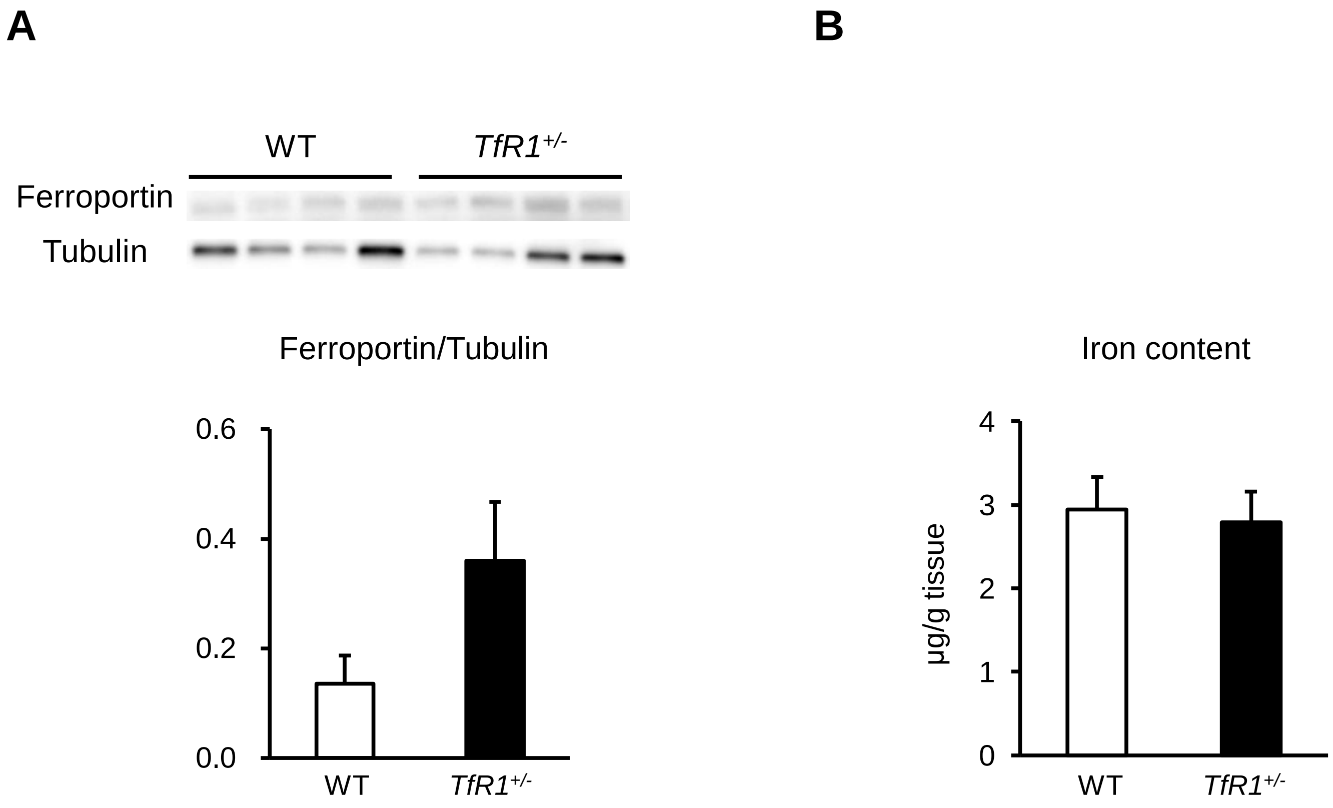

Haploinsufficiency of TfR1 on the development of angiogenesis in LI mice

(A) Representative western blotting image and summary densitometry graph illustrating expression of ferroportin and (B) iron contents in the ischemic adductor muscle of WT and *TfR1*$^{+/-}$ mice at 28 days after the surgery.

Unprocessed blot and gel images

The images displayed below are the unprocessed blot and gel figures used to generate the processed composite figures in the manuscript.

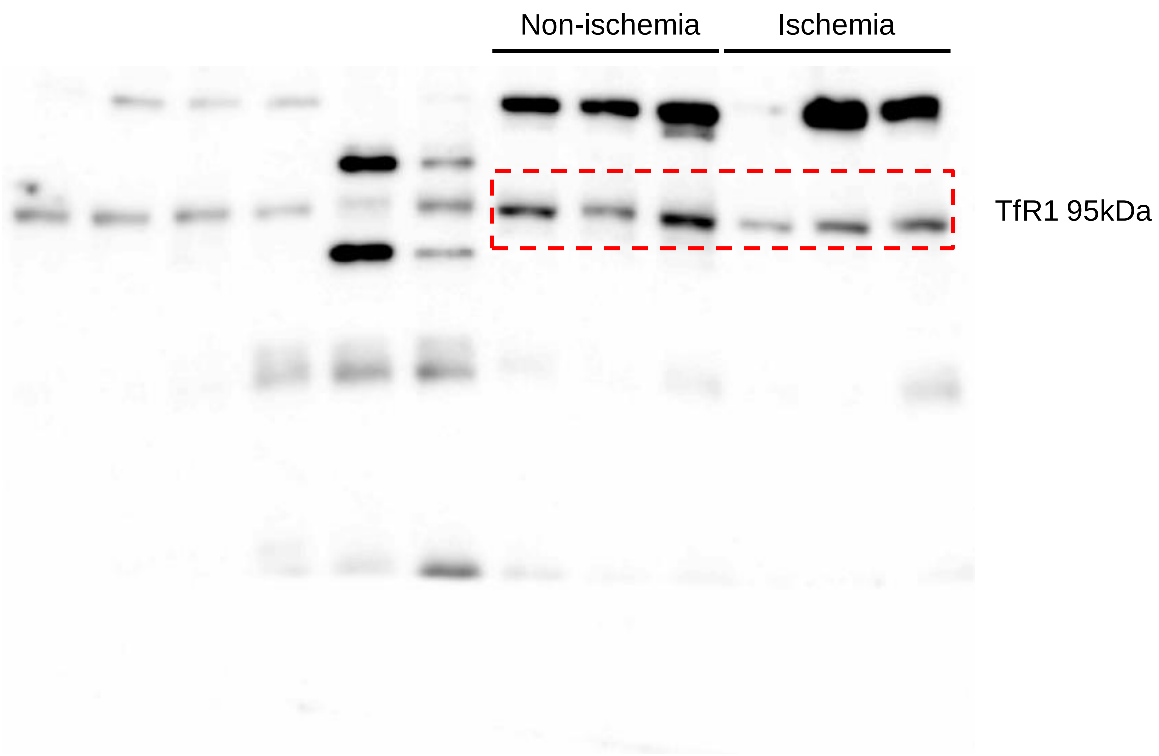

Original blots related to Figure 1B

Original Western blots of the cropped panels displayed in Figure 1B. Regions displayed in the main figure are indicated by dotted red lines.

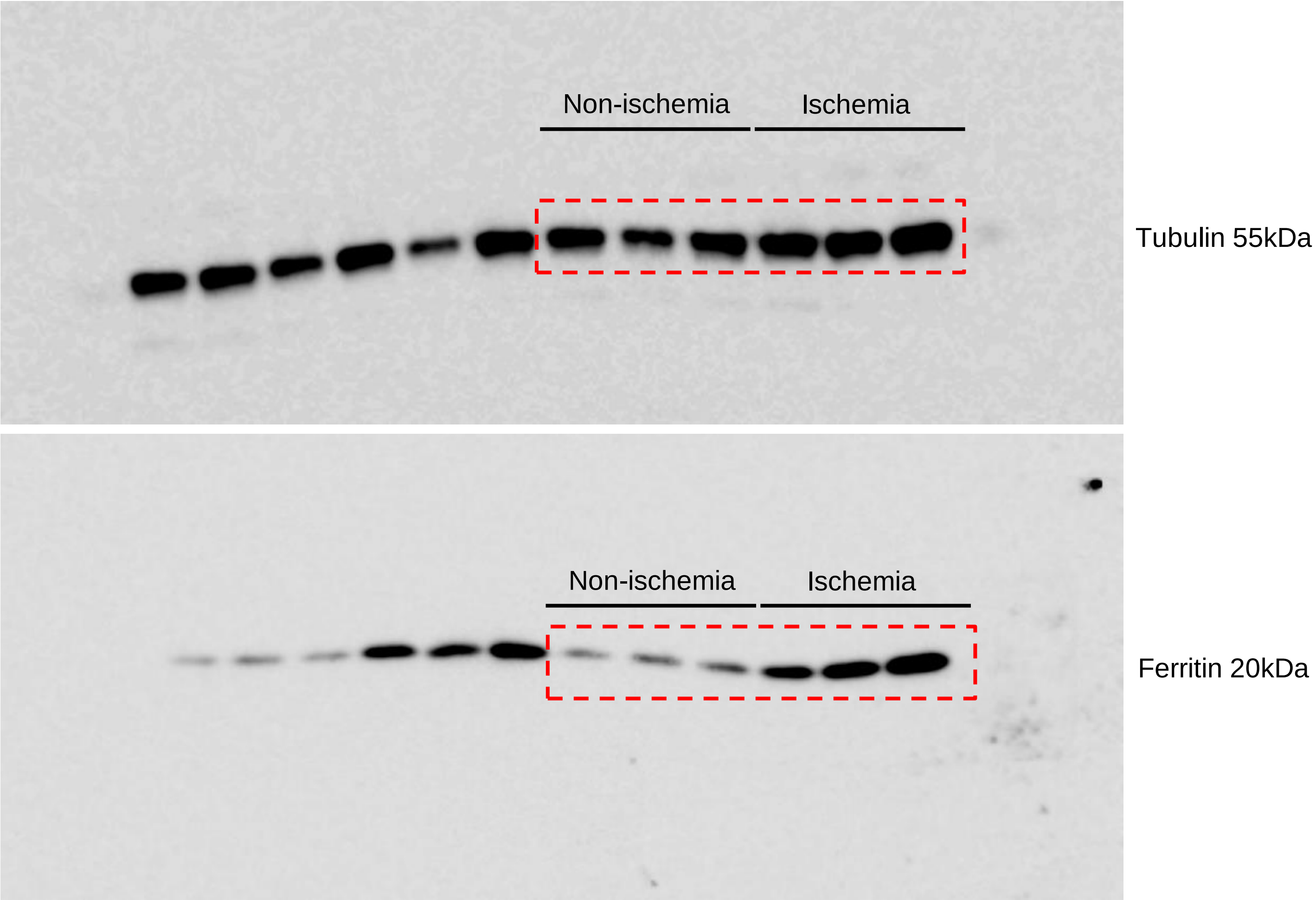

Original blots related to Figure 1B

Original Western blots of the cropped panels displayed in Figure 1B. Regions displayed in the main figure are indicated by dotted red lines.

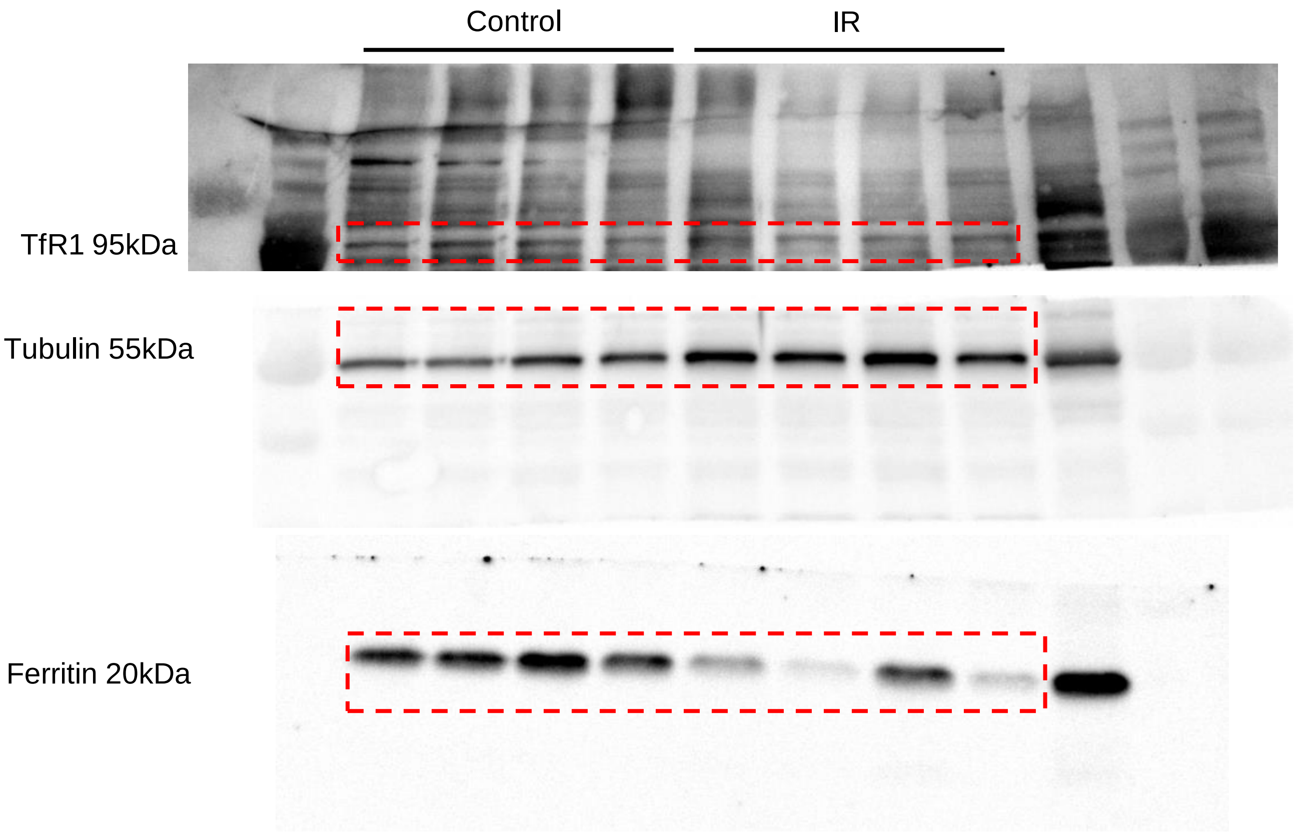

Original blots related to Figure 2A

Original Western blots of the cropped panels displayed in Figure 2A. Regions displayed in the main figure are indicated by dotted red lines.

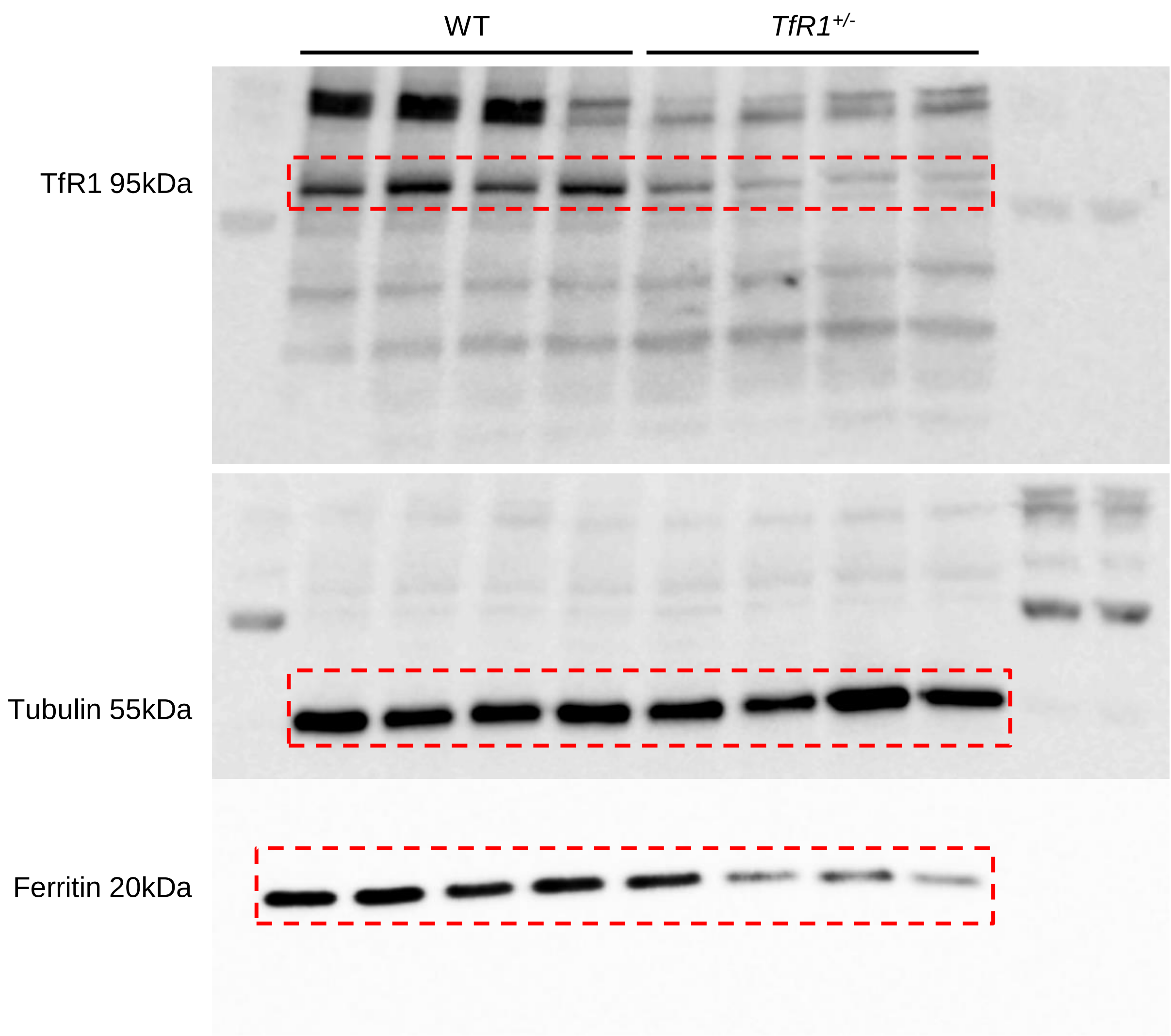

Original blots related to Figure 3A

Original Western blots of the cropped panels displayed in Figure 3A. Regions displayed in the main figure are indicated by dotted red lines.

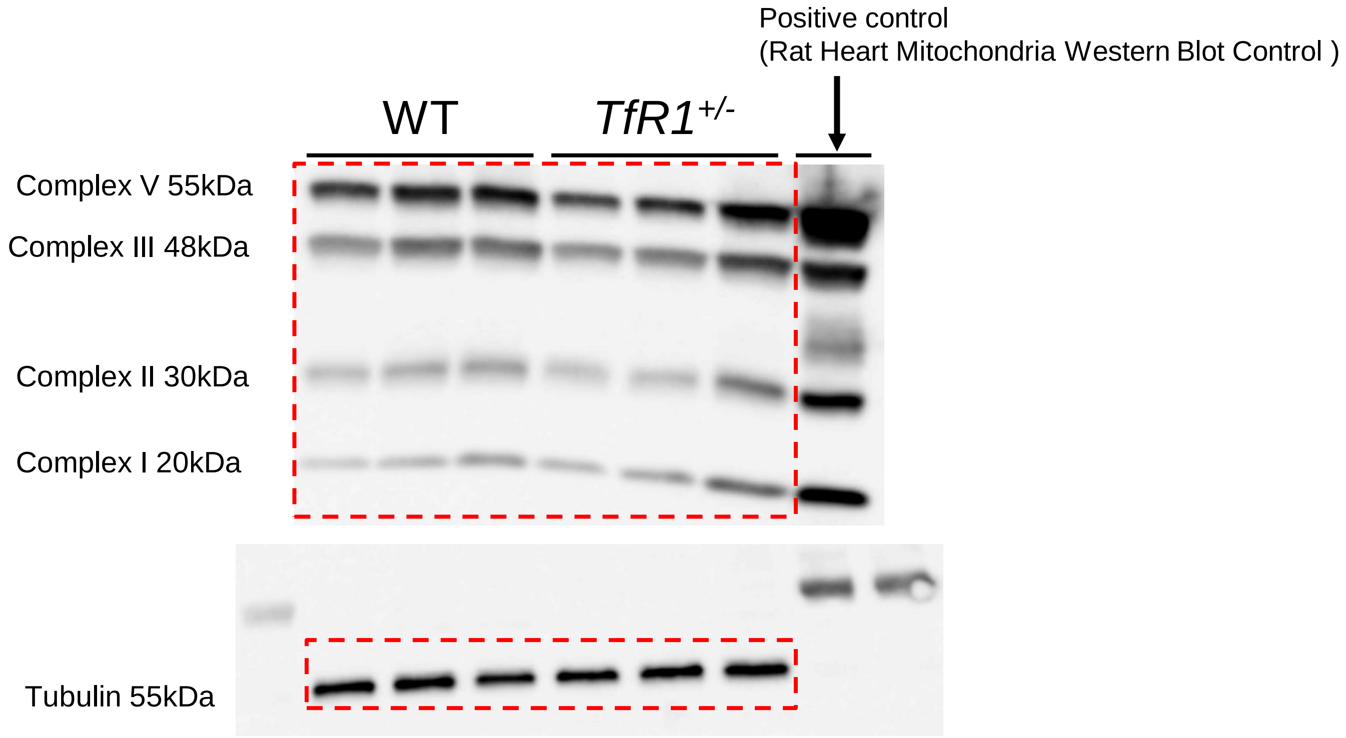

Original blots related to Figure 4A

Original Western blots of the cropped panels displayed in Figure 4A. Regions displayed in the main figure are indicated by dotted red lines.

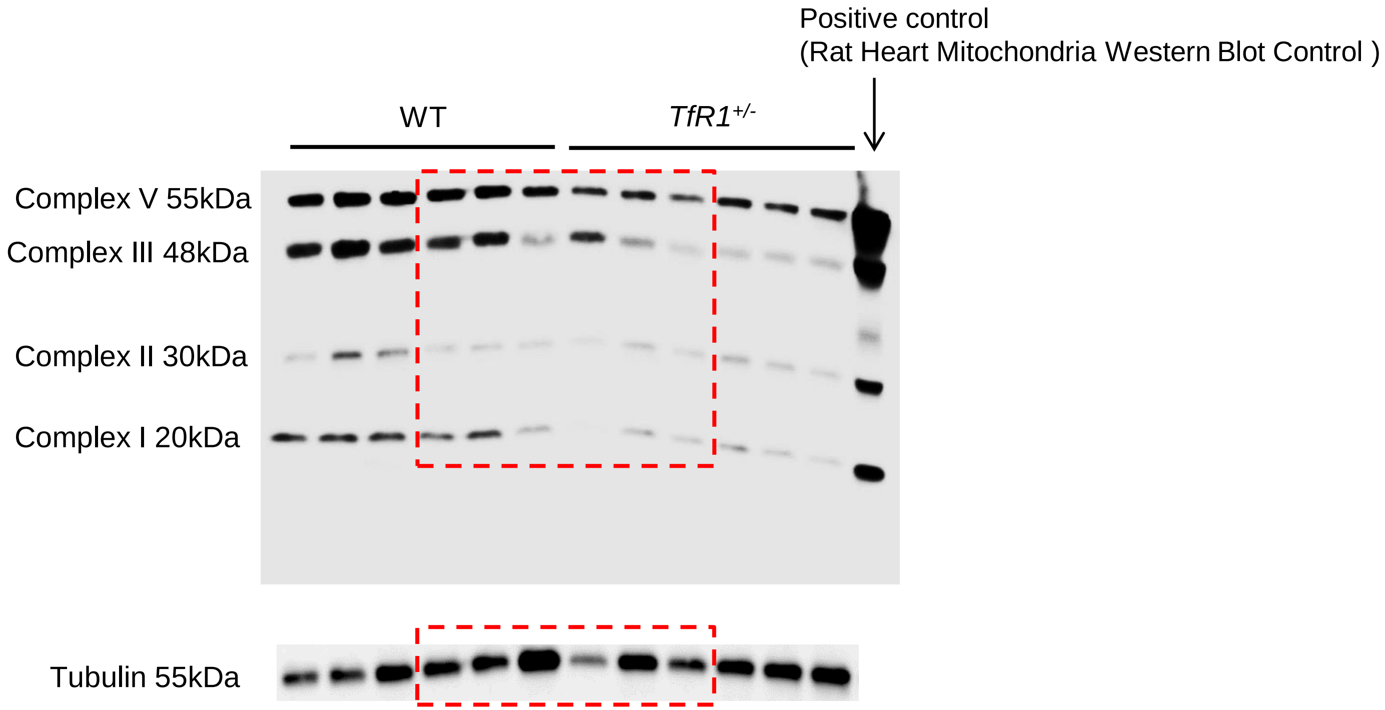

Original blots related to Figure 4B

Original Western blots of the cropped panels displayed in Figure 4B.
Regions displayed in the main figure are indicated by dotted red lines.

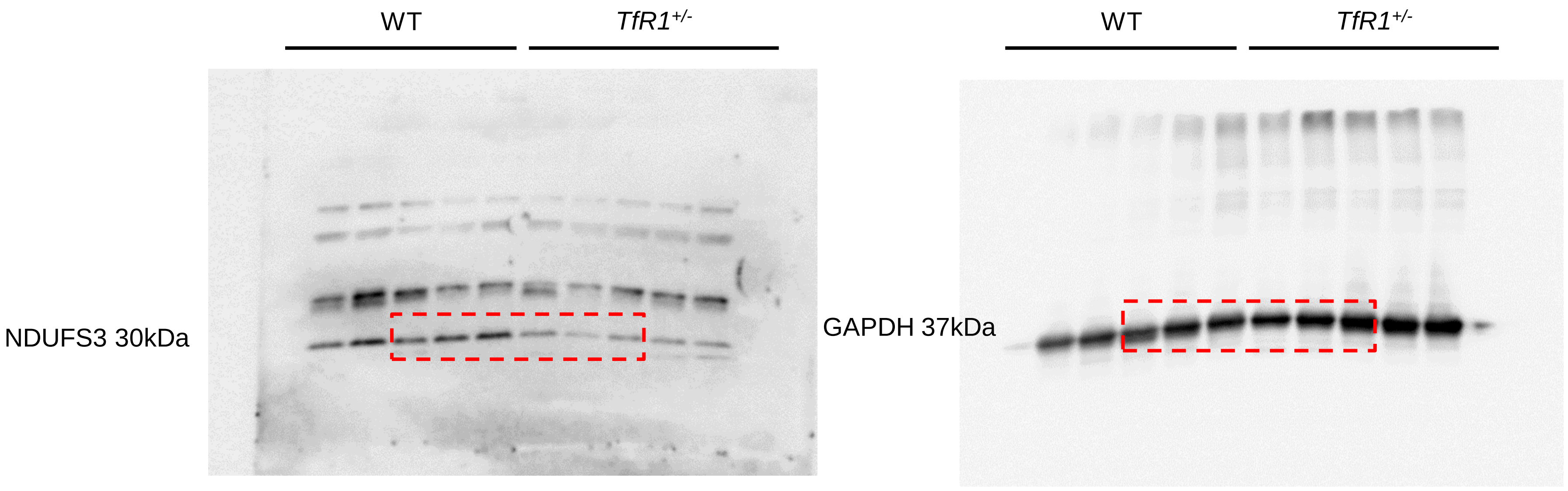

Original blots related to Figure 4C

Original Western blots of the cropped panels displayed in Figure 4C. Regions displayed in the main figure are indicated by dotted red lines.

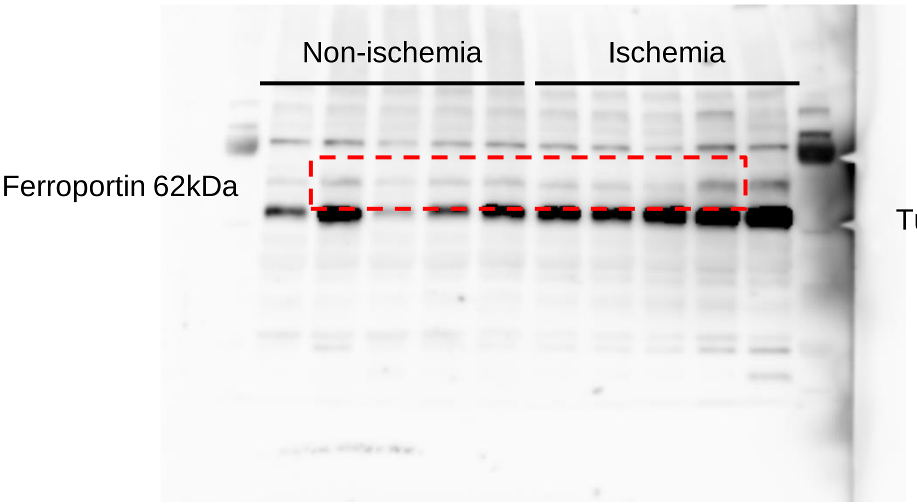

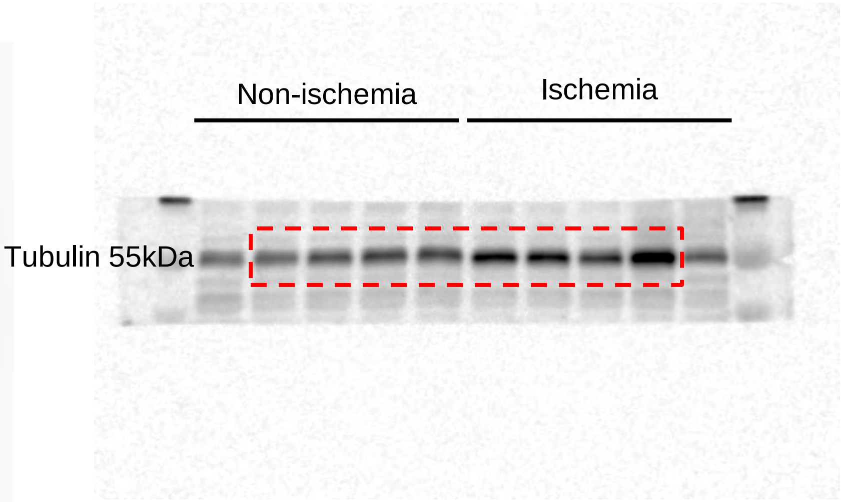

Original blots related to Supplementary Figure 1A

Original Western blots of the cropped panels displayed in Supplementary Figure 1A. Regions displayed in the main figure are indicated by dotted red lines.

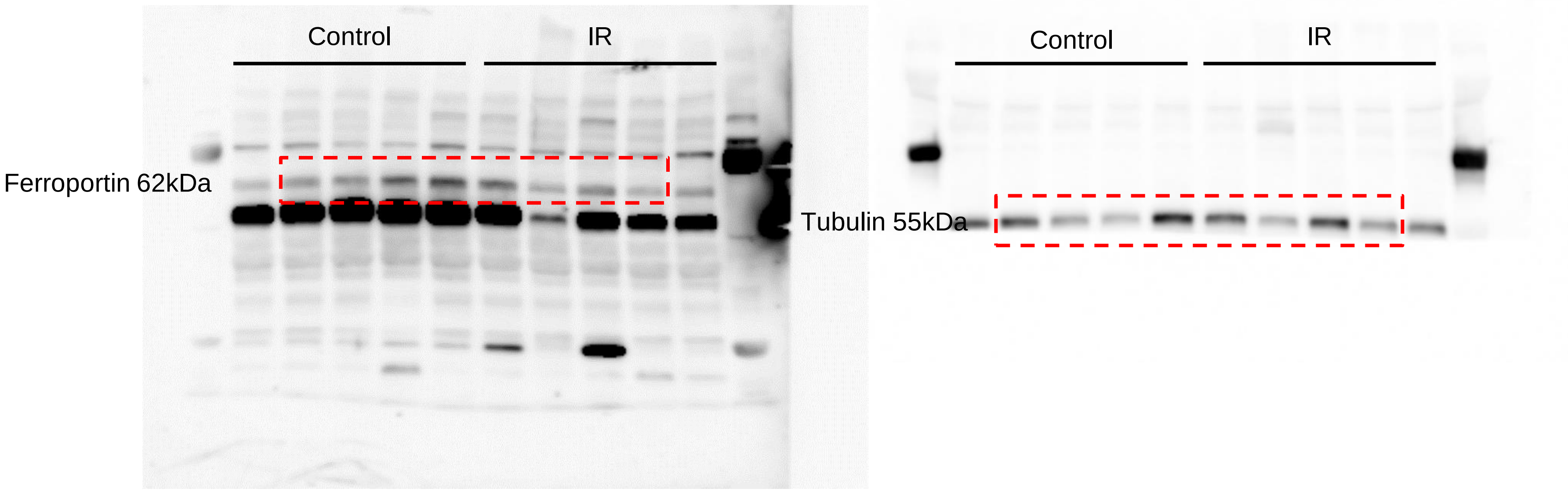

Original blots related to Supplementary Figure 2A

Original Western blots of the cropped panels displayed in Supplementary Figure 2A. Regions displayed in the main figure are indicated by dotted red lines.

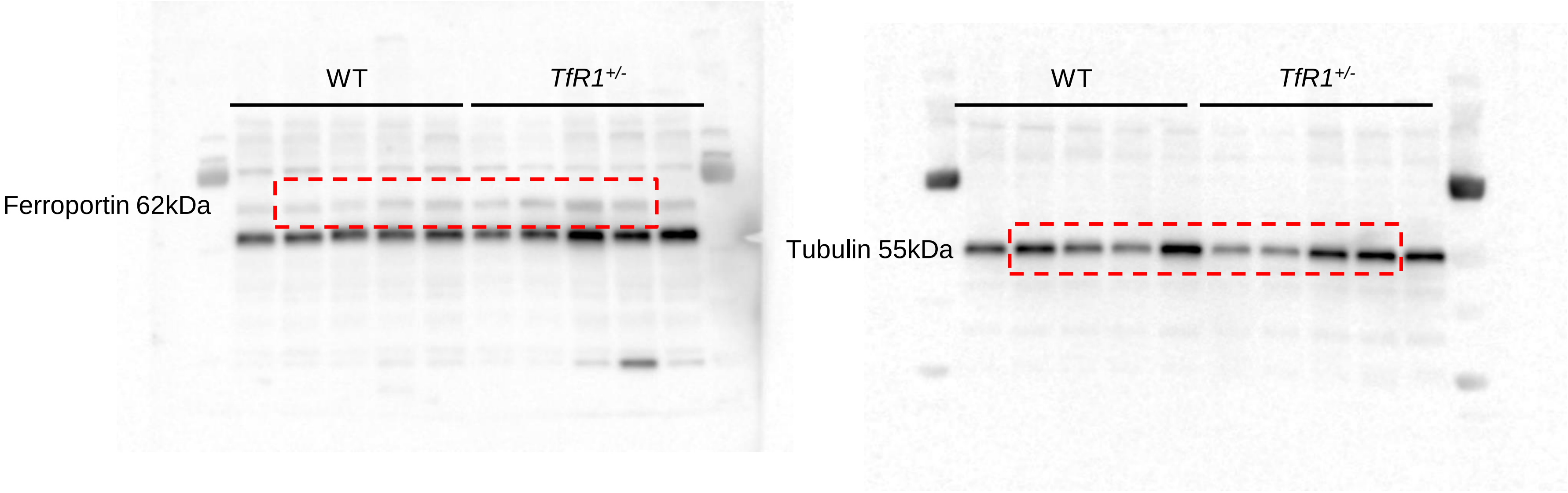

Original blots related to Supplementary Figure 3A

Original Western blots of the cropped panels displayed in Supplementary Figure 3A. Regions displayed in the main figure are indicated by dotted red lines.
